# The Treatment of Diphtheria

**Published:** 1889-04

**Authors:** 


### THE TREATMENT OF DIPHTHERIA.

In the last number of the Bulletin General de Therapeutique, Dr. G. Guelpa, of Paris, insisted on the value of irrigations of perchloride of iron solutions in diphtheria, and stated the following propositions:

1. Every case of diphtheritic angina not complicated with any other infectious affection, is followed, almost without exception, by recovery in the space of a week, if it be combated within the first twenty-four hours of its existence by irrigations in the throat and nares of a one-per-cent aqueous solution of perchloride of iron, and if these irrigations are practiced at least twice or three times every hour during both day and night.

2. In general, these irrigations in the throat prevent the extension of the diphtheria to the larynx and trachea.

3. In case of croup, especially if tracheotomy be early performed, pulverizations of the perchloride solution through the canula afford a good means of combating the disease, and increase greatly the chances of success.

Guelpa claims extraordinary results from this treatment, which, he says, must be carried out pitilessly in a disease so fraught with mortality. He dwells on the importance of the solution of ferric perchloride being weak, not concentrated. A solution of one per cent can do no harm if swallowed, or if it penetrate the larynx. Indeed, the latter contingency, according to this authority, is one rather to be sought for than avoided. There certainly, he says, is no danger that the ferric solution shall penetrate the air-passages below the larynx. The lavage of the larynx by the solution is one of the favorable conditions of the treatment; it is the only means of accomplishing the antisepsis of this organ, and it is the true preventive treatment of croup.

As for the means of making these irrigations, a common rubber ball injector with suitable nasal or mouth-piece is used. The child (in case the patient be a child) must be held firmly, the head being pressed between the left elbow and chest of the operator, the left hand of the latter being applied to the child's forehead, while, with the right, the canula of the irrigator is slipped into the mouth of the little patient. Advantage can sometimes be taken of the absence of a tooth for effecting this object. With the penetration of the canula, an effort of vomiting is generally excited. Then the operator presses upon the rubber ball, and the whole region of the fauces and pharynx is thoroughly and rapidly irrigated. The operation is done in a very few seconds, and without inflicting any suffering on the patient. But little of the injected liquid is swallowed, as the pharyngeal contraction which is excited expels most of it by the mouth. The nasal injections are less easily made than the buccal, but they are often indispensable, especially where there is a tendency on the part of the diphtheretic membrane to gain the nasal cavities. To prevent this untoward result, they may be given occasionally—every two or three hours.

Guelpa claims that his method is much easier of application than the swabbings or paintings of the throat so frequently practiced in diphtheria, or even than the administration of perchloride of iron mixtures by mouth at relatively short intervals.

It is needless to say that new modes of treatment of diphtheria have been the order of the day for the past forty years; but the method of Guelpa (who, by the way, is no mean authority on the subject) seems to have some measure of rationality in its favor, but the forcing a child to submit to such a process at short intervals will be an insuperable objection.—*Pac. Med. Journal.*

